# Grafted Human iPSC-Derived Neural Progenitor Cells Express Integrins and Extend Long-Distance Axons Within the Developing Corticospinal Tract

**DOI:** 10.3389/fncel.2019.00026

**Published:** 2019-02-12

**Authors:** Lindsey H. Forbes, Melissa R. Andrews

**Affiliations:** ^1^School of Medicine, University of St Andrews, St Andrews, United Kingdom; ^2^Biological Sciences, University of Southampton, Southampton, United Kingdom

**Keywords:** α9 integrin, induced pluripotent stem cell, transplantation, neural progenitor cell, sensorimotor cortex

## Abstract

After spinal cord injury (SCI), regeneration of adult motor axons such as axons in the corticospinal tract (CST) is severely limited. Alongside the inhibitory lesion environment, most neuronal subtypes in the mature central nervous system (CNS) are intrinsically unrepairable. With age, expression of growth-promoting proteins in neurons, such as integrins, declines. Integrin receptors allow communication between the extracellular matrix (ECM) and cell cytoskeleton and their expression in axons facilitates growth and guidance throughout the ECM. The α9β1 integrin heterodimer binds to tenascin-C (TN-C), an ECM glycoprotein expressed during development and after injury. In the mature CST however, expression of the α9 integrin subunit is downregulated, adding to the intrinsic inability of axons to regenerate. Our previous work has shown the α9 integrin subunit is not trafficked within axons of mature CST or rubrospinal tracts (RSTs). Thus, here we have utilized human induced pluripotent stem cell (iPSC)-derived neural progenitor cells (NPCs) to increase expression of α9 integrinwithin the developing rat CST. We demonstrate that human NPCs (hNPCs) express endogenous levels of both α9 and β1 integrin subunits as well as cortical neuron markers such as chicken ovalbumin upstream promoter transcription factor (COUP-TF) interacting protein 2 (Ctip2) and T-box brain 1 (Tbr1). In addition, lentivirus-mediated α9 integrin overexpression in hNPCs resulted in increased neurite outgrowth in the presence of TN-C *in vitro*. Following transplantation into the sensorimotor cortex of newborn rats, both wild type (WT) and α9-expressing hNPCs extend along the endogenous CST and retain expression of α9 throughout the length of the axonal compartment for up to 8 weeks following transplantation. These data highlight the growth potential of transplanted human iPSCs which may be a future target for regenerative therapies after nervous system injury.

## Highlights

– Increasing α9 integrin expression in human iPSC-derived NPCs increases neurite outgrowth *in vitro*.
– Human iPSC-derived NPCs transplanted into the rat developing sensorimotor cortex project axons within the endogenous CST.
– Exogenous expression of α9 integrin is retained within the axonal compartment of human iPSC-derived NPCs up to 8 weeks following transplantation.

## Introduction

Following injury to the mature central nervous system (CNS), the repair capacity is limited due to a myriad of intrinsic and extrinsic factors within the lesion site. After spinal cord injury (SCI), a characteristic glial scar densely packed with chondroitin sulfate proteoglycans (CSPGs) forms preventing further damage but also preventing regrowth of damaged axons (reviewed in Silver and Miller, 2004; reviewed in Tran et al., 2018). In addition, other inhibitory extracellular proteins including the myelin-associated protein, Nogo-A, are present and actively prevent regrowth (reviewed in Schwab and Strittmatter, 2014). Conversely, there is a downregulation of growth-promoting proteins, such as integrins (Bi et al., 2001; Condic, 2001; Franssen et al., 2015; reviewed in Nieuwenhuis et al., 2018) and Trk receptors (Lu et al., 2001), within mature CNS axons resulting in a poor neuronal regenerative response. Integrin receptors, known mediators of cell-cell and cell-matrix interactions, are transmembrane receptors comprised of one α and one β subunit. Within the developing CNS, integrins regulate cell cytoskeleton dynamics resulting in neurite outgrowth, axonal elongation and migration. Specifically, the α9 subunit, which forms a functional heterodimer with the β1 subunit (α9β1) binds tenascin-C (TN-C), the predominant extracellular matrix (ECM) glycoprotein in the CNS. Although several integrins including α9 are downregulated in the adult CNS, re-expression of integrins *in vitro* and *in vivo* rescues this inhibition resulting in increased axonal growth in the presence of inhibitory ECM proteins (Condic, 2001), including TN-C (Andrews et al., 2009; Cheah et al., 2016) which is secreted by reactive astrocytes. Recently, however, we have demonstrated that overexpressed integrin subunits (*via* viral vectors) are not transported within axons of the adult corticospinal or rubrospinal tract (CST and RST, respectively) *in vivo* (Andrews et al., 2016) presenting a challenge for gene therapy-mediated transmembrane receptor expression.

The field of regenerative medicine has also taken significant advantage of the recent discovery and development of induced pluripotent stem cell (iPSC) technology giving rise to infinite cell sources with high growth potential. Specifically, iPSCs and the numerous cell types which have successfully been derived from them, have great potential in the field of CNS regeneration whether through direct cell replacement and/or creation of a pro-regenerative environment (Nori et al., 2011; Lu et al., 2012, 2014; Tornero et al., 2013). In the current study, we use human iPSC-derived neural progenitor cells (hNPCs) as a vehicle to enhance α9 integrin expression within the CST following transplantation into the developing sensorimotor cortex. We show iPSC-hNPCs express a basal level of α9 integrin that can be augmented using lentiviral transduction. This overexpression leads to a significant increase in neurite outgrowth of cultured α9-hNPCs when grown on a TN-C substrate compared to controls. Following transplantation into the naïve sensorimotor cortex of neonatal rats, we demonstrate that both α9-hNPCs and wild type (WT) hNPCs survive for up to 8 weeks and extend axons within the CST reaching the pyramids within the medulla. Together these data highlight the ability of human iPSC-derived NPCs to develop and integrate within the rodent CNS as well as increase integrin activity within the CST that may contribute to future repair of the injured CNS.

## Materials and Methods

### Culture of Human iPSC-Derived NPCs

Human iPSC-derived NPCs (Axol Bioscience) were cultured as per manufacturer’s instructions with some modifications. Briefly, cells were cultured on 20 μg/mL poly-L-ornithine (PLO; Sigma) and 10 μg/mL laminin (Sigma) at a density of 5 × 10^4^/cm^2^. Cells were maintained in Neural Maintenance Medium (Axol Bioscience) and passaged using StemPro Accutase (Gibco™). For immunocytochemistry (ICC) analysis, cells were cultured on acid-washed glass coverslips coated with PLO and laminin as above.

### Production of 2nd Generation Lentivirus

Human embryonic kidney 293 cells expressing SV40 T antigen (HEK293T) producer cells were cultured in 10 cm plates (Nunc) and transfected with three 2nd generation plasmids [5 μg psPAX2, 2.1 μg pMD2.G-VSV.G (Naldini et al., 1996) and either 10 μg LV-PGK-α9-eYFP; (Andrews et al., 2016) or 6.5 μg LV-CMV-farnesylated green fluorescent protein (GFP) or (fGFP; Andrews et al., 2009)] using TransIT^®^ LT-1 transfection reagent (Mirus) at a ratio of 3:1 [reagent (μL):DNA (μg)]. The transfection mix was incubated with the producer cells for 24 h before being replaced with fresh complete DMEM [10% FBS (Seralab) and 1% Pen/Strep (Gibco™) in DMEM (Gibco™)]. Viral supernatant was collected from cells at 48 h and 72 h after initial transfection.

### Transduction of iPSC-Derived hNPCs

Seventy-two hours after thawing, iPSC-derived hNPCs (Axol Bioscience) were transduced with either LV-α9-eYFP or LV-fGFP supernatant, containing 2 μg/mL hexadimethrine bromide (polybrene, Sigma) for 4 h at 37°C. Following incubation, viral media was removed and cells were washed with Neural Maintenance Media, followed by incubation with fresh Neural Maintenance Media. Cells were cultured for a further 4–5 days before collection for transplantation, ICC analysis, or neurite outgrowth assays.

### Immunocytochemistry

Cells were fixed using 4% paraformaldehyde (PFA) and coverslips were washed in triplicate with PBS before being incubated with blocking solution (10% goat serum in PBS). Coverslips were incubated with primary antibodies diluted in blocking solution overnight at 4°C. The following primary antibodies were used: rabbit anti-brain-derived neurotrophic factor (anti-BDNF), rabbit anti-chicken ovalbumin upstream promoter transcription factor (COUP-TF) interacting protein 2 (anti-Ctip2), rabbit anti-doublecortin (anti-DCX), rabbit anti-glial fibrillary acidic protein (anti-GFAP), rabbit anti-myelin basic protein (anti-MBP), rabbit anti-T-box brain 1 (anti-Tbr1), rabbit anti-TN-C, rabbit anti-Tropomyosin receptor kinase B (anti-TrkB), rabbit anti-α9 integrin, mouse anti-β1 integrin, mouse anti-vinculin, mouse anti-βIII-tubulin and rabbit anti-βIII-tubulin (see Table 1 for concentrations). The following day, coverslips were incubated with secondary antibodies (at 1:1,000 dilution; goat anti-mouse AlexaFluor 488 or 568, goat anti-rabbit AlexaFluor 488 or 568; Invitrogen), and/or phalloidin (AlexaFluor 647 phalloidin; 1:250; Invitrogen) for 2 h. Cell nuclei were stained using DAPI (1:10,000; Thermo Scientific™) for 10 min before coverslips were mounted onto slides with FluorSave (Calbiochem) and imaged using a Leica D5500 epifluorescent microscope (Leica) with an attached DFC550 camera (Leica).

**Table 1:** Primary antibodies used for Immunocytochemistry (ICC), Immunohistochemistry (IHC) and Western blotting (WB).

Antibody	Species	Dilution			Supplier	Cat No.
		ICC	IHC	WB		
BDNF	Polyclonal, rabbit	1:200	-	-	Abcam	AB72439
Ctip2	Polyclonal, rabbit	1:500	-	-	Abcam	Ab70453
DAPI	-	1:10,000	1:10,000	-	Thermo Scientific™	62248
DCX	Polyclonal, rabbit	1:500	1:500	-	Abcam	AB18723
GFAP	Polyclonal, rabbit	1:750	1:500	-	DAKO	ZO33429-2
GFP	Polyclonal, rabbit	1:1,000	1:1,000	1:1,000	Invitrogen	A11122
HuNu	Monoclonal, mouse	-	1:500	-	Millipore	MAB1281
Iba-1	Polyclonal, rabbit	-	1:500	-	Wako	019–19741
MBP	Monoclonal, rabbit	1:50	-	-	Cell signaling	D8 × 4Q
hNCAM	Monoclonal, mouse	-	1:200	-	Santa Cruz, Insight	SC-106
PKCγ	Polyclonal, rabbit	-	1:200	-	Santa Cruz, Insight	SC-211
Tbr1	Polyclonal, rabbit	1:200	1:400	-	Abcam	AB31940
TN-C	Monoclonal, rabbit	1:250	1:250	-	Abcam	AB108930
TrkB	Polyclonal, rabbit	1:1,000	-	-	Santa Cruz Biotechnology	SC-12
α9-integrin	Polyclonal, rabbit	1:1,000	-	1:1,000	Thermo Scientific™	PA5–27771
β-actin	Monoclonal, mouse	-	-	1:10,000	Sigma	A5441
βIII-tubulin	Monoclonal, mouse	1:1,000	-	-	Sigma	T8660
βIII-tubulin	Polyclonal, rabbit	1:1,000	-	-	Covance	MRB-435P
β1-integrin	Monoclonal, mouse	1:500	-	1:1,000	BD Transductions	610467
Vinculin	Monoclonal, mouse	1:200	-	-	Millipore	MAB3574

### Western Blotting

Cell lysates were collected from hNPC cultures using 1× radioimmunoprecipitation assay (RIPA) buffer (containing 50 nM Tris, 150 mM NaCl, 1% NP-40 and 0.5% sodium deoxycholate) containing 25× complete protease inhibitor (Roche). Protein sample concentrations were determined using a bicinchoninic acid (BCA) assay kit (Thermo Scientific™) as per manufacturer’s instructions. Protein samples containing 40 μg protein diluted in water and NuPage loading dye were separated by electrophoresis on a 4%–12% bis-tris pre-cast gel (Invitrogen). Protein samples were denatured at 90°C for 10 min and were run alongside a PageRuler Plus Prestained protein ladder (Thermo Fisher). The gel was run with 1× NuPage MOPS SDS buffer and transferred to a nitrocellulose membrane (GELifesciences) with 1× NuPage transfer buffer. The membranes were blocked in 5% milk powder and stained overnight at 4°C with the following primary antibodies: rabbit anti-α9 integrin, mouse anti-β1 integrin, rabbit anti-GFP, and mouse anti-β-actin (see Table 1 for concentrations). The corresponding HRP secondary antibodies (HRP-mouse and HRP-rabbit, Invitrogen) were used at a dilution of 1:15,000 before analysis using enhanced chemiluminescence (ECL, GE Healthcare) and imaging with a LAS-3000 Lite imaging system. Images were processed and analyzed using ImageJ.

### Neurite Outgrowth Assays and Analysis

Neurite outgrowth assays were carried out on Permanox^©^ 8-well chamber slides (Thermo Scientific™). WT, α9-hNPCs and GFP-hNPCs were cultured on 20 μg/mL PLO and 10 μg/mL chicken TN-C (*n* = 4) or varying concentrations of human TN-C (Millipore): 1 μg/mL (*n* = 3), 5 μg/mL (*n* = 3) or 10 μg/mL (*n* = 3). Cells were plated at a density of 5 × 10^4^/well and incubated for 72 h at 37°C. The cells were fixed using 4% PFA and stained using the primary antibodies anti-GFP and anti-βIII-tubulin. Analysis and tracing of neurite outgrowth was performed using NeuronJ (Meijering et al., 2004; Schneider et al., 2012) and statistical analysis was performed using Microsoft Excel and Prism 5 GraphPad. A total of 30 neurite/axon lengths were measured for each condition. One replicate consisted of one vial of hNPCs cultured in one chamber slide (with 2 wells/condition). Data sets were analyzed using one-way analysis of variance (ANOVA) with Tukey *post hoc*.

### Preparation of Cells for Transplantation

For transplantation, WT hNPCs or α9-hNPCs were collected at day 8–9 in culture using StemPro Accutase, resuspended at a density of 1 × 10^6^/μL in Neural Maintenance Medium supplemented with Sure Boost (Axol Bioscience) and stored at RT until use.

### Transplantation of WT and α9-eYFP-Expressing hNPCs

All animal surgeries and procedures were conducted in accordance with the United Kingdom Animals (Scientific Procedures) Act 1986 under project licence number P48924394, and under the recommendations of the University of St. Andrews Welfare and Ethics Review Board and UK Home Office guidelines. The protocol was approved by the University of St. Andrews Animal Welfare and Ethics Review Board and UK Home Office. Newborn Sprague-Dawley rats received transplants of either WT hNPCs (*n* = 26) or α9-eYFP-expressing hNPCs (*n* = 17). Rat pups between the age of postnatal day 0–2 (P0–P2) were anesthetized on ice for 3–5 min. Pups were transferred to a surgical platform containing ice to maintain anesthesia. For surgery, each pup was secured using a custom-made 3D-printed rat pup head frame (kindly printed by Dr. Robert Hammond, Univ of St Andrews). Using stereotaxic coordinates, each pup received either a unilateral or bilateral transplant in two specific sites with 1 × 10^6^ cells per target site (1 μL/injection) into layer 5 of the neonatal sensorimotor cortex. Coordinates that produced the most on-target injections were: AP 0.0 mm, ML 1.5/−1.5 mm, DV −0.4 mm. Cells were injected manually using a Hamilton syringe with a custom-made 30 gauge stainless steel needle over the course of 1 min with the needle left in place for a further 2 min to prevent backflow. Following surgery, pups were transferred to a heated cage to recover before being returned to their mother. On-target injections included pups where a noticeable scar was evident within the target site. All pups with off-target injections were excluded from the study.

### Tissue Perfusion and Sectioning

Tissue was collected at the following time points: 2 weeks (*n* = 9); 4 weeks (*n* = 7*); 6 weeks (*n* = 9); 7 weeks (*n* = 10) and 8 weeks (*n* = 8; Table 2; * indicates animals at 4 weeks from WT group only). Any rats with off-target injections are not included in Table 2. Rats were administered a terminal dose of sodium pentobarbital and transcardially perfused with 0.9% saline solution (pH 7.4) and 4% PFA (pH 7.4). The brain and spinal cord were removed from each animal, post-fixed in 4% PFA for 48–72 h and cryoprotected in 30% sucrose solution for a further 48–72 h. Tissue was cryo-sectioned in either the coronal or sagittal plane using a sliding microtome (Leica) at a thickness of 40 μm. Sections were stored in 0.05% sodium azide in PBS at 4°C prior to processing for immunohistochemistry (IHC).

**Table 2:** Tissue analysis time points.

	Tissue collection time points					
	2 weeks	4 weeks	6 weeks	7 weeks	8 weeks	Total
No. of pups with WT hNPC grafts	6	7	4	6	3	26
No. of pups with α9-hNPC grafts	3	0*	5	4	5	17
Total	9	7	9	10	8	43

*Only two animals received on target injections in this group and have therefore been removed from the analysis.

### Immunohistochemistry

Sections were incubated in blocking solution for 2 h at RT and then with primary antibody overnight at 4°C. Primary antibodies used were as follows: rabbit anti-DCX, rabbit anti-GFAP, rabbit anti-human nuclear antigen (anti-GFP), mouse anti-HuNu, mouse anti-human neural cell adhesion molecule (anti-hNCAM), rabbit anti-TN-C and rabbit anti-Tbr1 (see Table 1 for full details). Tissue sections were incubated with the corresponding secondary antibodies (all at a dilution of 1:750; goat anti-mouse AlexaFluor 488 or 568, goat anti-rabbit AlexaFluor 488 or 568; Invitrogen) for 2 h at RT. All nuclei were stained using DAPI (1:10,000; Thermo Scientific™). Tissue sections were then mounted onto 0.25% gelatin-coated slides and coverslipped using FluorSave (Calbiochem). Slides were imaged using a Leica D5500 epifluorescent microscope (Leica) with an attached DFC550 camera (Leica). Images were processed and collated using ImageJ and Microsoft Powerpoint.

## Results

### Human iPSC-Derived NPCs Express Growth-Promoting Proteins *in vitro*

**Figure 1:**
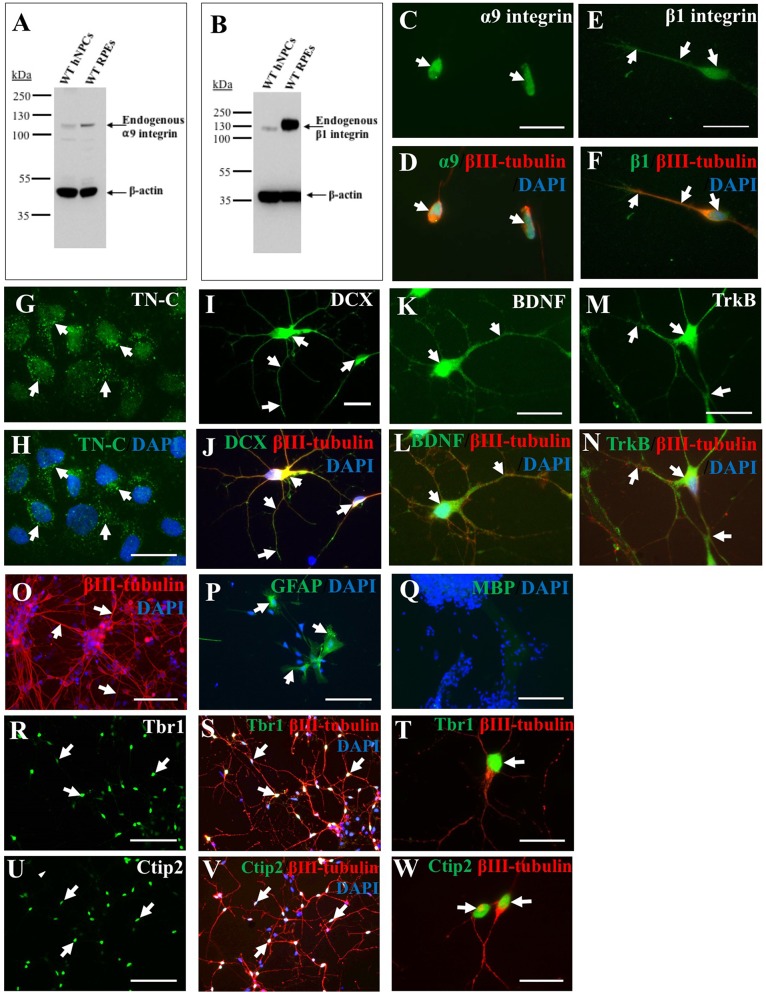
Endogenous expression of integrins, growth-promoting proteins and deep layer cortical neuron markers in human induced pluripotent stem cell (iPSC)-derived neural progenitor cells (NPCs). Endogenous α9 **(A)** and β1 **(B)** integrin subunit expression in wild type (WT) human NPCs (hNPCs) was confirmed using western blotting (WB), with faint bands observed at approximately 115 kDa (α9 integrin) or 120 kDa (β1 integrin). This was analyzed alongside retinal pigmented epithelial (RPE) cell lysates as positive controls for both integrin subunits. Blots were also stained with β-actin (approximately 42 kDa) to confirm equal protein loading. Immunocytochemistry (ICC) analysis further confirmed a low level of endogenous α9 **(C,D)** and β1 **(E,F)** integrin subunit expression within hNPCs. Furthermore, ICC analysis demonstrated hNPCs express tenascin-C (TN-C) as observed in punctate dots (white arrows, **G,H**) around the cell nucleus (identified using DAPI). The hNPCs also express a number of growth-promoting proteins including the progenitor cell marker doublecortin (DCX), observed in both the cell body and neurite projections (white arrows, **I**), which co-labeled with βIII-tubulin (white arrows, **J**). Expression of the neurotrophic factor brain-derived neurotrophic factor (BDNF; white arrows in **K,L**) was also identified as well as expression of its receptor, Trk B (white arrows in **M,N**). The hNPC cultures largely contained βIII-tubulin-positive cells (white arrows in **O**), however some glial fibrillary acidic protein (GFAP)-positive cells (white arrows in **P**) were also detected. No myelin basic protein (MBP)-positive cells were detected following 21 days in culture **(Q)**. Expression of deep-layer cortical neuron markers T-box brain 1 (Tbr1; white arrows, **R–T**) and chicken ovalbumin upstream promoter transcription factor (COUP-TF) interacting protein 2 (Ctip2; white arrows, **U–W**) was detected following ICC within the DAPI-stained nucleus of hNPCs. Cells were co-stained with βIII-tubulin and DAPI. Scale bar in (**C–N,T,W**) = 20 μm; (**O–S,U,V**) = 100 μm.

A primary goal of this study was to characterize iPSC-derived hNPCs *in vitro* by analyzing expression of neuronal and growth-promoting proteins as well as neurite outgrowth capacity on TN-C. To analyze endogenous integrin expression (α9 and β1 subunits) within the hNPCs, both western blotting (WB) and ICC were used. WB data indicated expression of both α9 (Figure 1A) and β1 (Figure 1B) integrin subunits in iPSC-derived hNPCs *in vitro* which was further confirmed by ICC (Figures 1C–F). Interestingly, cultured hNPCs also express an endogenous level of TN-C (Figures 1G,H), the ligand for the α9β1 heterodimer, a finding previously shown with PC12 cells (Andrews et al., 2009).

Alongside integrin expression, further ICC analysis revealed iPSC-derived hNPCs express the progenitor cell marker DCX (Figures 1I,J) and the growth-promoting protein BDNF (Figures 1K,L) and its receptor TrkB (Figures 1M,N). The hNPCs used within these experiments were pre-programmed to differentiate into cerebral cortical neurons (Shi et al., 2012b), however further cell types could be detected within the culture including a small proportion of GFAP-positive cells (approximately 5%) indicative of astrocytes (Figure 1P). No cells were positive for MBP following 21 days in culture (Figure 1Q). The majority of the culture, however was positive for βIII-tubulin (approximately 95%; Figure 1O) indicating a high proportion of cells had a neuronal phenotype. To confirm previous literature (Shi et al., 2012b) for this cell population, the hNPCs were analyzed for the expression of deep layer cortical neuron markers Ctip2 and Tbr1, with a high proportion (approximately 70%) of the cell population expressing these proteins within the cell nucleus (Figures 1R–W).

### Increasing α9 Integrin Expression in hNPCs Results in Increased Neurite Outgrowth in the Presence of TN-C *in vitro*

**Figure 2:**
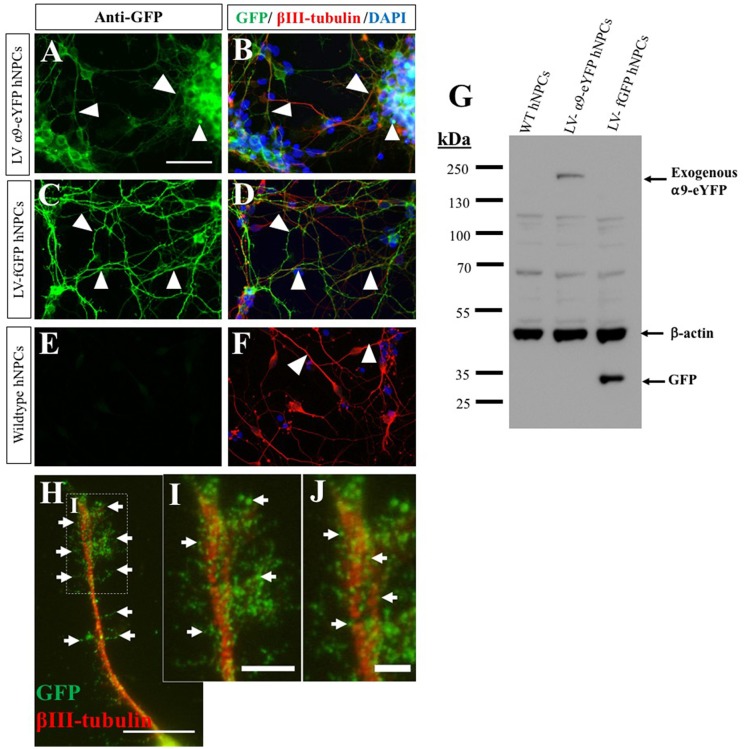
Overexpression of α9-enhanced yellow fluorescent protein (α9-eYFP) and farnesylated green fluorescent protein (fGFP) using lentivirus. The overexpression of α9-eYFP was detected using ICC with an anti-GFP antibody (white arrows in **A**) detected within the hNPC projections (white arrows in **B**) and co-labeled with βIII-tubulin. Integrin expression was observed in the cell body and throughout the length of the projections. fGFP expression was identified following hNPC transduction using LV-fGFP detected using GFP and βIII-tubulin antibodies (white arrows in **C,D**). No GFP expression was observed in untransduced WT hNPCs **(E,F)**. Overexpression was confirmed using WB **(G)** with an anti-GFP antibody, resulting in a band of approximately 140 kDa in lane 2 (LV-α9-eYFP hNPC lysates) and a band at approximately 30 kDa in lane 3 (LV-fGFP hNPC lysates). No GFP bands were observed in lane 1 containing WT hNPC lysates. β-actin immunoblotting confirmed equal protein loading (approximately 42 kDa). At high magnification, α9-eYFP was observed co-localizing to the tip of extended projections in a punctate arrangement (white arrows in **H–J**). Scale bar in **(A–F)** = 50 μm, **(H)** = 15 μm, **(I)** = 5 μm and **(J)** = 2.5 μm.

In addition to demonstrating endogenous expression of the α9 integrin subunit, we also overexpressed α9 integrin (tagged to eYFP) within iPSC-derived hNPCs using lentiviral transduction, resulting in an approximate 90% transduction efficiency. As a control, expression of fGFP was used in addition to untransduced WT hNPCs. Following ICC analysis for anti-GFP, expression of both α9-eYFP and fGFP were observed within the cell bodies and neurites of the hNPCs (Figures 2A–F), which was further confirmed by WB (Figure 2G).

In order for cells to migrate and axons to extend as well as interact with the extracellular environment, integrins need to be enriched at nascent focal adhesion sites. This is known as integrin clustering, a process that prompts intracellular signaling which coincides with cytoskeletal reorganization. Following overexpression of α9-eYFP within hNPCs, the extending neurites displayed dense enrichment of α9-eYFP *in vitro* (Figures 2H–J). Furthermore, localization of α9 integrin-eYFP was observed in vinculin- and actin-rich focal adhesion sites (Supplementary Figure S1). In addition, we observed a subtle enhancement of β1 integrin expression upon overexpression of α9 integrin in hNPCs, evaluated by western blot (Supplementary Figure S2). This suggests that α9 overexpression may lead to increased α9β1 heterodimer formation and/or upregulation of β1 integrin subunits.

**Figure 3:**
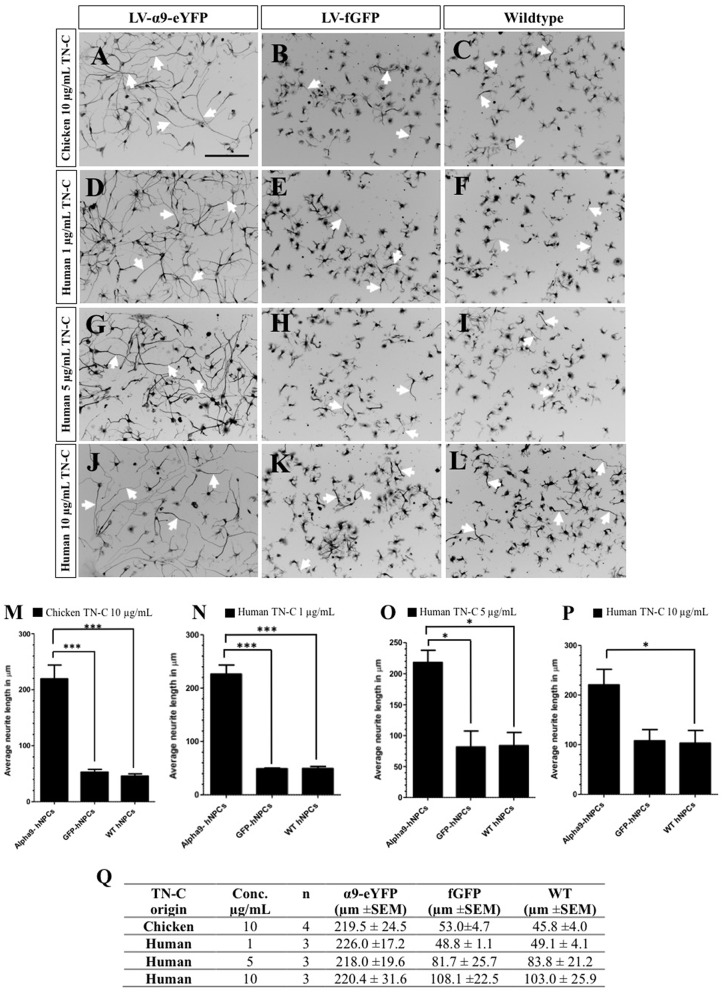
Increasing expression of α9 integrin in iPSC-derived hNPCs promotes neurite outgrowth when grown on TN-C. Following transduction of hNPCs with either LV-α9-eYFP or LV-fGFP, cells were grown on either 10 μg/mL chicken TN-C (*n* = 4; **A,-C,M,Q**) or 1 μg/mL (*n* = 3), 5 μg/mL (*n* = 3) or 10 μg/mL (*n* = 3) of human TN-C **(D–L,N–Q)**. Cells were fixed after 72 h in culture and analyzed with ICC using anti-βIII-tubulin to label neurites (white arrows in **A–L**) which were measured using NeuronJ. Results were analyzed using one-way analysis of variance (ANOVA) and expressed as mean ± SEM. *P < 0.05, ***P < 0.001. Scale bar in **(A–L)** = 200 μm.

The function of exogenously expressed α9-eYFP protein in hNPCs was analyzed using a neurite outgrowth assay in the presence of either chicken or human TN-C, at varying concentrations after 72 h in culture. As previously shown, inducing α9 integrin expression in PC12 cells stimulates neurite outgrowth in the presence of TN-C, an inhibitory growth substrate for cells lacking α9 integrin (Andrews et al., 2009). On chicken TN-C (10 μg/mL), a significant difference in neurite outgrowth was observed for LV-α9-hNPCs compared to both fGFP-expressing hNPCs (*P* < 0.001) and WT hNPCs (*P* < 0.001; Figures 3A–C,M,Q). A small amount of neurite outgrowth was observed from the two control groups grown on TN-C (Figures 3B,C,M), likely due to low levels of endogenous α9 and β1 integrin expression (Figure 1). To determine whether the hNPCs showed any specificity for human TN-C, neurite outgrowth was analyzed on varying concentrations of human TN-C (1 μg/mL, 5 μg/mL and 10 μg/mL). Results from these assays show an increase in neurite outgrowth from the LV-α9-hNPCs as compared to the other groups most significantly with 1 μg/mL human TN-C (Figures 3D–L,N–Q) further highlighting the ability of α9 expression to increase outgrowth in the presence of TN-C. However, as the concentration of human TN-C was increased to 5 μg/mL and 10 μg/mL, the outgrowth from both the fGFP-hNPCs and WT hNPCs also increased, while the neurite outgrowth in the α9-hNPCs group did not increase further. Specifically, neurite outgrowth of fGFP-hNPCs grown on 1 μg/mL human TN-C was found to be 48.8 μm ± 1.1 (*n* = 3), WT hNPCs 49.1 μm ± 4.1 (*n* = 3) and α9-eYFP-hNPCs 226.0 ± 17.2 (*n* = 3), whereas with 10 μg/mL human TN-C, the outgrowth from both fGFP-hNPCs and WT hNPCs more than doubled to 108.1 μm ± 22.5 and 103.0 μm ± 25.9, respectively, whilst outgrowth from α9-hNPCs remained relatively constant at 220.4 μm ± 31.6 (Figures 3O–Q).

### Human iPSC-Derived NPCs Transplanted Into the Developing Rat Sensorimotor Cortex Project Within the Intrinsic Pyramidal Tract

We next assessed the ability of hNPCs to survive, express exogenous growth-promoting integrin receptors and extend axonal processes following transplantation into the rat neonatal sensorimotor cortex. Regeneration of the adult CST, one of the main pathways governing motor control, has been shown to have particularly limited regenerative capacity (reviewed in Tuszynski and Steward, 2012). We recently demonstrated that axonal localization of growth-promoting integrins, including α9, is hindered within the mature CST compared to the developing CST and sensory pathways (Andrews et al., 2016).

**Figure 4:**
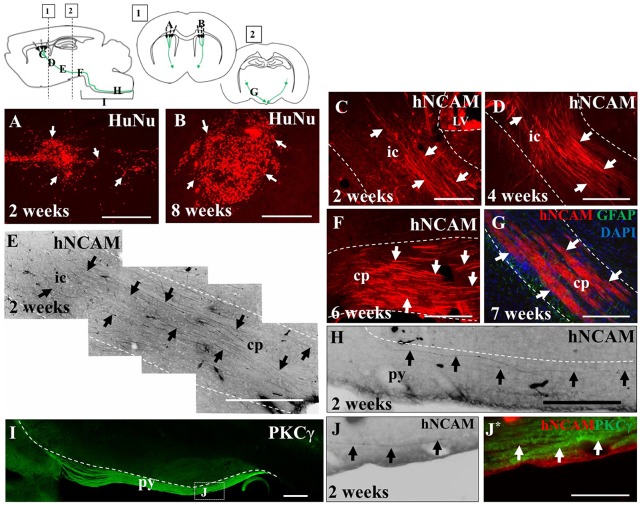
iPSC-derived hNPCs injected into the sensorimotor cortex extend axons along the intrinsic pyramidal tract, projecting to the brain stem. Tissue was stained with anti-human nuclear antigen (anti-HuNu) which detected the cell bolus at the injection site which was either dispersed (white arrows in **A**) or more compact (white arrows in **B**). The projections were identified using anti-human neural cell adhesion molecule (anti-hNCAM) and were observed emanating from the bolus through areas of the intrinsic pyramidal tract. These included the internal capsule **(C–E)**, the cerebral peduncles, **(F,G)** and areas of the pyramids (H,J,J*). To confirm that the fibers observed at 2 weeks post-transplantation within the brain stem were within the pyramidal tract, tissue was stained for protein kinase C gamma (PKCγ), to detect the pyramids (I,J,J*). Abbreviations: ic, internal capsule; cp, cerebral peduncle; py, pyramidal tract. Scale bar in **(A,B,F,G)** = 250 μm, **(C,H)** = 200 μm; **(D,E,I)** = 500 μm; (J,J*) = 150 μm.

In this study, pups aged between P0–P2 received grafts of either WT hNPCs or α9 hNPCs into layer 5 of the sensorimotor cortex and tissue was analyzed at 2, 4, 6, 7 and 8 weeks post-transplantation. Injections that were off-target, specifically where no part of the hNPC bolus was observed within the target site, have been excluded from the study. This was determined by identifying the deep layers of the sensorimotor cortex which was immunopositive for Tbr1-expressing cells (Supplementary Figure S3). Grafts were detected using human-specific antibodies for HuNu, to identify the nuclei of the hNPCs (Figures 4A,B), and hNCAM to identify the projections emanating from the graft site (Figures 4C–H,J,J**). In 20.9% of pups (*n* = 9 out of the total 43), most of which were from the later time points (6, 7 and 8 weeks), no surviving hNPCs were detected and instead an injection scar was present at the intended injection site, identified by an upregulation of GFAP staining (data not shown).

**Figure 5:**
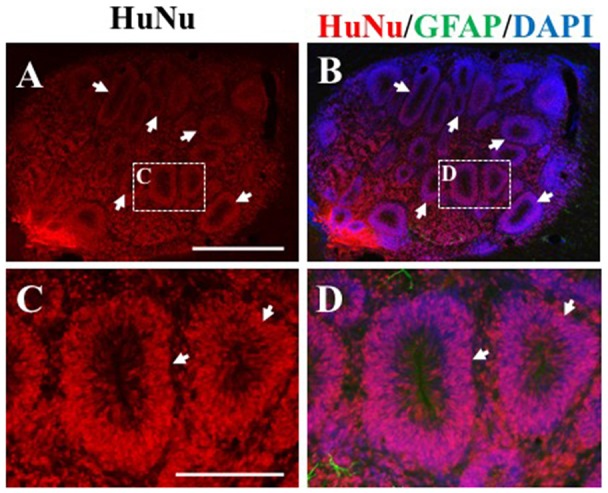
Rosette formation within HuNu-positive hNPC bolus. Following transplantation, some hNPCs formed a rosette formation (white arrows, **A–D**), typical of neural stem cells (NSCs). Scale bar **(A,B)** = 250 μm; **(C,D)** = 150 μm.

In the remaining 79.1% of pups (*n* = 34), graft survival of iPSC-derived hNPCs into the sensorimotor cortex was observed from 2 weeks up to 8 weeks. Across all time points, the formation of the cell bolus varied from one which spread out (Figure 4A) to a bolus that was more densely packed (Figure 4B). In some grafts, rosette formation was observed, a common feature of neural stem cells (NSCs; Figure 5; Shi et al., 2012a). In addition, there was an extensive number of hNCAM-positive axonal projections observed within regions of the intrinsic corticospinal pathway including the internal capsule, cerebral peduncles and areas of the brain stem, specifically in the pons and pyramids (Figure 4). Furthermore, there was variation in thickness of hNCAM-positive projections. Some projections appeared very narrow and thin (Figures 4C,D), whilst others appeared not as single fibers but as thicker bundles (Figure 4G) suggesting fibers may have bundled together over time. In following the trajectory of the hNCAM-positive axonal projections at the 2 week time point, axonal projections were found within the pyramids, specifically within the protein kinase C gamma (PKCγ)-positive region used to identify the pyramidal tract (Figure 4I; Bradbury et al., 2002). More specifically, when analyzing WT hNPCs grafts, a total of 3.8% (*n* = 1) of WT hNPCs showed projections terminated within the medullary pyramids. Similarly, a total of 3.8% (*n* = 1) of grafts showed projections terminated within the pons. A further 23.1% (*n* = 6) of grafts showed hNCAM-positive projections as far as the cerebral peduncles whilst 34.6% (*n* = 9) showed projections reaching the internal capsule. In 23.1% (*n* = 6) of grafts, projections were only found emanating within the localized region of the cell bolus within the cortex, whilst a further 11.5% (*n* = 3) showed no NCAM-positive projections due to potential degeneration of the cell bolus. Interestingly, we did not find projections reaching the pyramids at later time points (4, 6, 7, or 8 weeks).

**Figure 6:**
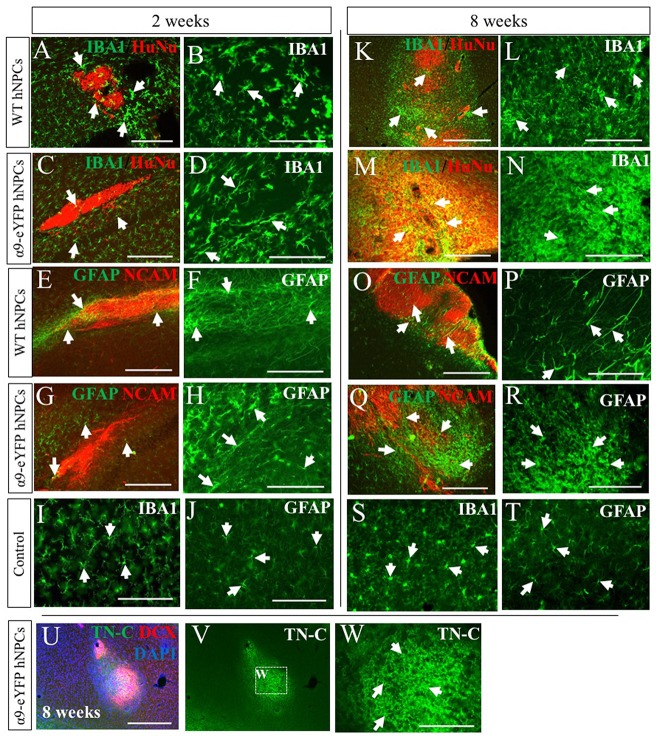
Transplantation of human iPSC-derived NPCs results in an increased rat host immune response and expression of TN-C over time. Tissue surrounding the graft sites was analyzed using IHC for expression of IBA-1 and GFAP (indicated by white arrows in A-T) alongside either HuNu and hNCAM to identify the graft site. At early time points of 2 weeks (**A,C**—low magnification; **B,D**—high magnification), IBA-1 staining is mildly increased compared to control tissue **(I)**. However, over time there is a noticeable increased immune response from microglia surrounding the HuNu-positive graft site (**K,M**—low magnification; **L,N**—high magnification) compared to control tissue **(S)**. Similarly, GFAP staining is mildly increased at earlier time point of 2 weeks (**E,G**—low magnification; **F,H**—high magnification) compared to control tissue **(J)**. Over time this expression is markedly increased (**O,Q**—low magnification; **P,R**—high magnification) compared to control tissue **(T)**. Following transplantation of hNPCs there was a localized upregulation of TN-C at the graft site **(U–W)**. Scale bar in **(A,C,E,G)** = 250 μm; **(B,D,F,H–J,L,N,P,R–T,W)** = 150 μm; **(K,M,O,Q,U,V)** = 500 μm.

As this was a xenogeneic transplant, the host immune response was analyzed following grafting of hNPCs using an antibody against IBA-1, a marker for microglia, the main immune cell of the CNS and against GFAP, to identify astrocytes which can become reactive with an immune response. At early time points of 2 weeks (Figures 6A–D), microglial activity was increased slightly compared to control tissue (contralateral hemisphere, Figure 6I) for both WT and α9-eYFP hNPC grafts. Over time however, microglial activity further increased demonstrated with a clear increase in positive IBA-1 staining at the graft site (Figures 6K–N). GFAP immunoreactivity showed a similar pattern of activity for astrocytes surrounding the graft site. At 2 weeks post-transplantation, GFAP expression was mildly increased (Figures 6E–H) compared to control tissue (Figure 6J) and by 8 weeks this was markedly increased (Figures 6O–R,T) indicative of reactive astrocytes at the graft site. Furthermore, there was no indication that the small population of astrocytes (~5%) that were initially present in the hNPC cultures contributed to the GFAP-immunoreactivity with minimal to no overlap in staining between transplants and GFAP (Supplementary Figure S4). The expression of the ECM protein TN-C within the tissue was also analyzed following transplantation of both WT hNPCs and α9-eYFP-expressing hNPCs. Similar to the staining observed with GFAP and IBA-1, expression of TN-C was increased at the site of transplant across all time points (Figures 6U–W).

**Figure 7:**
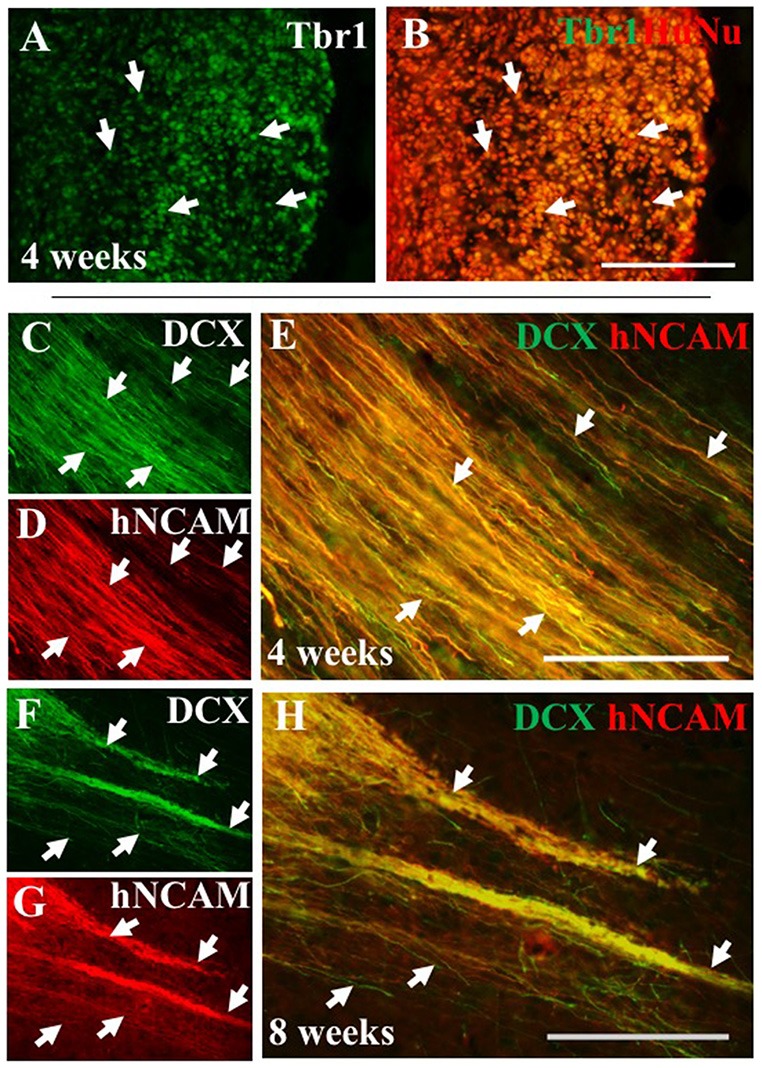
iPSC-derived hNPCs express deep-layer cortical neuron markers and progenitor cell markers *in vivo*. The expression of the cortical neuron marker Tbr1 was analyzed using an antibody against Tbr1 demonstrating that a large proportion of the transplanted hNPCs expressed Tbr1 that co-localized within HuNu-positive cells at 4 weeks (**A,B**, white arrows). This expression was observed in both WT and α9-eYFP hNPCs across all time points (data not shown). Similarly, expression of the progenitor cell marker DCX was retained following transplant shown at 4 weeks **(C–E)** and at 8 weeks **(F–H)** post-transplantation within hNCAM-positive projections (white arrows **C–H**). Scale bar in **(A–H)** = 150 μm.

To further characterize iPSC-hNPC transplants *in vivo*, expression of cortical neuron markers were assessed. We have shown cortical neuron markers were expressed *in vitro* prior to transplantation (Figure 1). IHC analysis confirmed this expression was maintained following transplantation, with a large proportion of the cells retaining their cortical identity and expressing the deep-layer cortical neuron marker, Tbr1, from 2 weeks up until 8 weeks (the longest time point analyzed; Figures 7A,B). Furthermore, we also investigated whether these cells matured or remained immature over the 2 month time period of analysis, using the progenitor cell marker DCX. The hNPCs retained DCX expression as long as the cells were present suggesting that at least some of the cells remained in a progenitor cell state over a period of 2 months post-transplantation (Figures 7C–H). When endogenous expression of rat DCX was analyzed within the cortex, expression was high at 2 weeks postnatally/post-transplant, yet declined thereafter (data not shown).

### Exogenously Expressed α9 Integrin Is Retained in Transplanted hNPCs and Localized Throughout the Axonal Compartment *in vivo*

**Figure 8:**
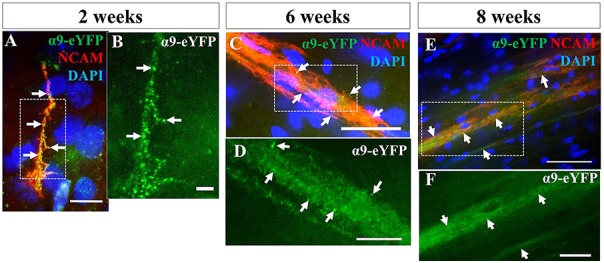
iPSC-derived hNPCs retain exogenous α9 integrin expression up to 8 weeks *in vivo*. Following IHC analysis with an anti-GFP antibody, exogenous α9-eYFP expression was detected from 2 weeks up to 8 weeks. This expression co-localized throughout hNCAM-positive axonal projections **(A,C,E)**. At 2 weeks α9-eYFP expression was observed as punctate dots throughout the axons **(A,B)**. Scale bar in **(A)** = 5 μm; **(B)** = 2.5 μm; **(C)** = 25 μm; **(D)** = 10 μm; **(E)** = 50 μm; **(F)** = 20 μm.

We next investigated the extent to which axons from transplanted α9-expressing hNPCs could project within a developing neonatal system whilst maintaining integrin expression within axons. Analysis of α9-eYFP hNPC grafts identified that α9-eYFP was expressed throughout the projected axons in small punctate dots (Figure 8) at each time point from 2 weeks to 8 weeks indicating axonal expression, transport and/or targeting of this exogenous protein.

**Figure 9:**
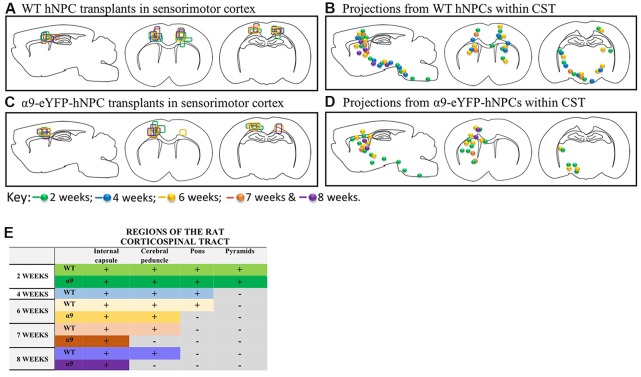
In the uninjured developing rodent brain, increasing integrin expression does not result in longer projections from iPSC-derived hNPCs. Transplants from all 43 pups were compared within the two groups (WT and α9-eYFP hNPC transplants). The transplant sites for both groups (panels **A,C**) indicate that both groups received on-target injections across all time points (2 weeks to 8 weeks). When comparing axonal projections from these two groups (panels **B,D,E**), results show the distance axons of WT hNPCs project are comparable to the distance α9-eYFP hNPCs project. At 2 weeks, both groups show projections emanating through the pyramidal tract reaching the pyramids. Similarly, at 4 weeks, WT hNPCs projected axons from the cortex to the pons (the α9-eYFP hNPCs at 4 weeks have been removed from analysis due to small group size). At 6 weeks, WT hNPCs were also able to project to the pons whilst α9-eYFP hNPCs projected only as far as the cerebral peduncles. Finally, at 7 and at 8 weeks, WT hNPC projections were detected within the cerebral peduncles and α9-eYFP hNPCs were detected only as far as the internal capsule.

Further analysis of α9-eYFP hNPC grafts confirmed that fibers projecting from these cells followed the intrinsic CST from the sensorimotor cortex through the internal capsule, cerebral peduncles and pons. Specifically, the transplanted α9-hNPCs reached the pyramids by 2 weeks post-transplantation in the best case, comparable to WT hNPCs (Figure 9). Interestingly at later time points, similar to WT hNPCs, axons projecting from these cells did not project as far as they had at 2 weeks post-transplantation and in many cases α9-hNPCs did not project axons as far as WT hNPCs (Figure 9). For example, at 6 weeks, WT hNPC projections were identified within the pons whereas α9-eYFP hNPCs only reached the cerebral peduncles. Similarly, at 7 weeks post-transplantation, WT hNPCs projected axons to the cerebral peduncles while α9-eYFP hNPCs only projected as far as the internal capsule (Figure 9). These results suggest that endogenous levels of integrins are sufficient to induce significant levels of axonal growth likely because the nervous system as well as the transplanted cells are still undergoing development. In further analysis of α9-eYFP hNPC grafts, a total of 5.9% (*n* = 1) showed projections within the medullary pyramids. No projections were observed within the pons, yet 11.8% (*n* = 2) of grafts showed hNCAM-positive projections within the cerebral peduncles. In 29.4% (*n* = 5) of the grafts, there were projections within the internal capsule whilst in 17.6% (*n* = 3) of the grafts there were projections only within the localized cortical region of the cell bolus. A further 35.3% (*n* = 6) showed no hNCAM-positive projections likely due to the degeneration of the cell bolus.

## Discussion

Stem cell research has radically changed regenerative medicine, even more so since the discovery of iPSC technology (Takahashi and Yamanaka, 2006; Takahashi et al., 2007). In addition, we now have valuable insight into the underlying mechanisms that prevent and contribute to regeneration of human axons following CNS injury. Specifically, it has been shown that the failure of CST axon regeneration is partly due to a loss of anterograde trafficking of growth-promoting proteins including the α9 integrin subunit as demonstrated in cultured cortical neurons (Franssen et al., 2015). The present study demonstrates that hNPCs can increase expression of α9 integrin within the developing rodent CST. We have carried out extensive characterization of these cells both *in vitro* and following transplantation into the developing sensorimotor cortex. These cells express low levels of the integrin subunits, α9 and β1, as well as other growth-promoting proteins including BDNF and TrkB, and the cortical neuron markers Tbr1 and Ctip2. Furthermore, we have generated α9-eYFP-expressing hNPCs and demonstrated the functional effect of the α9 receptor in the presence of TN-C *in vitro*. Following transplantation, hNPCs retained expression of α9-eYFP up to 8 weeks post-transplantation, the time point after which the transplanted cells died. After only 2 weeks post-transplantation, we demonstrate that these cells project axons within the endogenous CST reaching as far as the medullary pyramids. Due to the growth-promoting properties of α9-eYFP, we initially hypothesized that α9-eYFP-expressing hNPCs would project longer axons than WT hNPCs, however the presence of α9-eYFP did not result in longer axonal projections *in vivo* compared to WT hNPCs. These results suggest that endogenous levels of integrins are sufficient to induce axonal growth from hNPCs within a developing nervous system.

### Endogenous Protein Expression in hNPCs

A large proportion of the cultured hNPCs were βIII-tubulin-positive confirming a high proportion of neurons within these cultures. Although these cells were pre-programmed to differentiate into cerebral cortical neurons, other cell types such as GFAP-expressing cells were identified within the cell population suggesting that these hNPC cultures may give rise to a small proportion (~5%) of astrocytes. For the purposes of transplantation, the presence of astrocytes may have been advantageous for hNPC survival and integration *in vivo*. For example, astrocytes can promote neuron function and synaptogenesis (Ullian et al., 2001, 2004; Christopherson et al., 2005).

It was also critical that the expression profile of the cells have a cortical neuron phenotype which is important for their development, integration and function (Finger et al., 2002; Arlotta et al., 2005; Sano et al., 2017) and because we were transplanting these cells into the cortex these proteins are required to induce growth within the CST. Specifically, when transplanting cells into the lesioned cortex, research has shown that receiving an area-specific graft can impact the level of integration and regeneration observed (Gaillard et al., 2007; Michelsen et al., 2015). Previous literature has shown expression of cortical neuron markers within iPSC-hNPCs, such as Tbr1 and Ctip2 (Shi et al., 2012b), which our results confirm *in vitro*. Specifically, these proteins are involved in projection of neurons from deep-layers of the cortex to sub-cortical targets (Hevner et al., 2001; Arlotta et al., 2005; Chen et al., 2008; McKenna et al., 2011). Of particular interest is Ctip2, which is known to be involved in formation of CST projections during development while perturbation of its expression can result in aberrant CST formation (Chen et al., 2008). Following transplantation, we were also able to confirm hNPCs retained Tbr1 expression over time. Due to a lack of consistency with Ctip2 antibodies *in vivo*, however, we were unable to clearly ascertain Ctip2 expression in hNPCs following transplantation. Evaluating integrin expression *in vitro*, specifically of α9 and β1 integrin, we have also demonstrated that iPSC-derived hNPCs express low levels of each integrin subunit which can be further enhanced using lentiviral transduction (as shown for α9 integrin) *in vivo*.

### Overexpressing α9-eYFP Results in Increased Neurite Outgrowth From hNPCs in the Presence of TN-C

Overexpression of α9-eYFP in hNPCs was successfully achieved using lentivirus. We used full-length α9 integrin tagged to eYFP to allow the exogenous protein to be detected *in vivo* using an anti-GFP antibody due to a lack of commercially available α9 integrin antibodies that can detect this integrin subunit *in vivo*. The function of α9 integrin was analyzed using a neurite outgrowth assay in the presence of TN-C as previously described (Andrews et al., 2009). Our results show neurite outgrowth from α9-eYFP hNPCs was significantly greater compared to either fGFP-hNPCs or untransduced WT hNPCs when grown on chicken TN-C (10 μg/mL). The α9-eYFP hNPCs also had significant outgrowth on low concentrations of human TN-C (1 μg/mL) suggesting they may show greater specificity for human TN-C. Interestingly, when the concentration of the human TN-C was increased to 5 μg/mL and 10 μg/mL, the outgrowth from both the fGFP-hNPCs and WT hNPCs gradually increased compared to their growth on 1 μg/mL TN-C, whilst the outgrowth α9-eYFP hNPCs plateaued. These results suggest endogenous integrin function is increased as the concentration of human TN-C is increased.

Research has shown embryonic dorsal root ganglia neurons (DRGs) can increase integrin cell surface expression in the presence of inhibitory CSPGs resulting in integrin-induced outgrowth (Condic et al., 1999). This adaptation is not mimicked with adult neurons, however overexpression of integrin in adult DRG neurons can rescue this effect and result in increased neurite growth (Condic, 2001; Cheah et al., 2016). Our present results suggest that increasing the concentration of human TN-C results in an intrinsic activation of endogenous integrin function. Although other TN-C-binding integrin subunits such as α7 and α8 (Mercado et al., 2004) may influence neurite outgrowth, expression of these subunits was not assessed within this study but furthermore they have not been directly linked to neurite outgrowth.

### Characterization of hNPC Transplants in Developing Rat Sensorimotor Cortex

We used a neonatal rat model and transplanted cells between the ages of P0–2 into the predicted layer 5 of the sensorimotor cortex using a developmental rat brain atlas (Altman and Bayer, 1995). Coordinates were optimized with each new litter and all off-target grafts were rejected from study analysis. The coordinates that resulted with the most on-target transplants were: AP 0.0 mm, ML 1.5/−1.5 mm, DV −0.4 mm. A window of opportunity exists at early postnatal ages where the rat immune system is not yet fully developed (reviewed in Marshall-Clarke et al., 2000; reviewed in Holsapple et al., 2003) allowing for longer term survival of grafts that ensures analysis, compared to a naïve adult rodent model. Transplantation at this age also exploits the advantages of a readily developing nervous system which may facilitate integration and development of cells and their axonal projections. Transplantation of embryonic stem cell (ESC)-derived NPCs and neurons has previously been carried out in the immune-competent rodent neonatal brain with promising results for graft survival and integration (Ideguchi et al., 2010; Denham et al., 2012). For example, transplanted ESC-derived neurons can survive up to 10 weeks following transplant into the neonatal rat striatum (Denham et al., 2012). Similarly, ESC-derived NPCs transplanted into P0–P2 mouse cerebral cortex differentiated into area-specific cortical projection neurons and projected within the host CST within 2–3 weeks after transplantation (Ideguchi et al., 2010). In the present study, transplant survival was highest at earlier time points of 2 and 4 weeks, after which IHC results show an increase in Iba-1 immunoreactivity indicating microglia activity (or infiltration) and an increase in GFAP immunoreactivity indicating astrocytic activity, correlating with a decrease in graft survival in conjunction with a loss of axonal projections. In addition, despite the increase in GFAP expression at the transplant site, there was minimal colocalization between transplanted cells and GFAP immunoreactivity confirming the fact that any transplanted astrocytes (up to 5% total of the cultured hNPCs prior to transplant) did not significantly contribute to the increase in astrocytes over time.

Using human-specific antibodies, the transplanted cell bolus and emanating projections were detected *in vivo*. Specifically, these projections followed the trajectory of the CST through the corona radiata and internal capsule, cerebral peduncle, pons and pyramids of the brain stem. The furthest projections were observed within the pyramids 2 weeks following transplantation, in both WT hNPC and α9-eYFP hNPC transplants. By 8 weeks, WT hNPCs were only found to project as far as the cerebral peduncles and α9-eYFP hNPCs were found to project only as far as the internal capsule. It is unclear whether at this time point, the axons were actively dying back in conjunction with the increased immune response but the increase in microglial activation suggests this may have been the case.

It is important to consider not only the survival and projections of transplanted cells, but also whether the cells integrate and mature *in vivo*. Over a 2 month time period, we would expect the cells to develop and follow a path of maturation, which was assessed by analyzing levels of DCX expression. Previously published work on DCX expression following transplantation of mouse embryonic cortical neurons show DCX expression is reduced 2 weeks following transplant into the adult lesioned motor cortex (Ballout et al., 2016). However, rodent cells and human cells follow different time courses of development and maturation. In the human NPCs used in this study, DCX expression was retained from 2 weeks up until 8 weeks *in vivo* suggesting that a large proportion of the cells are maintained in a progenitor cell state following grafting. The expression of endogenous DCX was present in the developing rat cortex at 2 weeks at a very low level and by 4 weeks, no DCX expression was detected. This is in line with previously published literature suggesting cell proliferation ends at around P15 (reviewed in Rice and Barone, 2000). We also assessed vesicular glutamate transporter 1 (vGLUT1) expression in the cultured hNPCs and found it to be expressed throughout the cells. We did not investigate expression following transplantation since the growing axons did not reach their targets in the spinal cord, however this is an area for future investigation.

Another way to analyze hNPC maturation is by assessing myelination. Research has suggested hNPCs must mature and become functional neurons before myelination can occur (Lu et al., 2014). Following immunolabeling for MBP, we were unable to confirm myelination of hNPCs *in vivo* (data not shown) however it may be advantageous for hNPCs to retain an immature cell state when considering the need to extend long distance axonal projections and make new synaptic contacts. Recent research has demonstrated human iPSC-derived cortical neurons (Axol Bioscience) require long-term culture of 20–30 weeks for functional synapses to form (Odawara et al., 2016). Similarly, transplanted human NSCs into the adult immunodeficient rodent lesioned spinal cord showed no signs of maturation until 3 months *in vivo* with oligodendrocyte formation not detected until 1 year post-transplant (Lu et al., 2017). These results highlight the requirement for long-term analysis of human iPSC-NPCs *in vivo* to enable full characterization of integration and maturation.

### Why Do α9-eYFP hNPC Axonal Projections Not Extend Further Than WT hNPCs *in vivo*?

Over the period of 8 weeks, α9-eYFP hNPC-derived axons did not project as far as the WT hNPC-derived axons (Figure 9). There are a number of potential reasons which may have impacted on this outcome. Previous literature suggests genetic modification of transplanted cells using lentivirus does not affect cell viability, providing the host is immunocompromised (Behrstock et al., 2008; Fujimoto et al., 2012). Some studies however have reported a significant loss of virally-transduced cells following engraftment into the rodent brain. For example, transplantation of mouse luciferase-expressing NSCs into immune-competent adult mouse brain show 80% of cells undergo apoptotic cell death within the first 24 h following grafting, with only 1% of cells surviving 14 days following transplant. This was correlated with an increase in hypoxic marker expression within the graft bolus suggesting lack of oxygen and nutrients contributed to reduced cell survival (Reekmans et al., 2012).

As the ligand for α9β1 is TN-C, the expression of this ECM protein was analyzed within the endogenous tissue and was found to be localized to the area surrounding the transplant sites following engraftment of hNPCs (Figure 6). It is unclear whether the localized expression of TN-C at the injection site limited the hNPCs projections from extending long distances. If α9-eYFP-hNPCs preferentially project to a TN-C rich environment this may predict their behavior if transplanted into an injury model, where TN-C is upregulated at the injury site (Zhang et al., 1997). Although care was taken during the transplant, the injection itself may have induced a micro-injury that could have impacted upon α9 integrin activity. The results from the outgrowth assays show α9-eYFP was functional prior to transplantation yet proteins associated with CNS injury, including CSPGs and Nogo-A, inactivate integrin activity (Hu and Strittmatter, 2008; Tan et al., 2011). Modulation of the integrin activation state using forced expression of kindlin-1 or human-specific antibodies TS2/16 (Hu and Strittmatter, 2008; Tan et al., 2011; Cheah et al., 2016), can overcome this inhibition and may be required if α9-eYFP-hNPCs were to be used for regenerative purposes in the CNS.

### Trafficking of α9 Integrin Within the CST

We know some integrin subunits, including α9, are not efficiently transported into the axons of certain neuron subtypes including adult CST axons (Andrews et al., 2016). Recent work by Franssen et al. (2015) have highlighted that Rab11- and Arf6-mediated trafficking of integrins play a key role in their age-related downregulation as a result of increased retrograde integrin trafficking leading to a reduced presence of integrins within the axonal compartment. However, during development, integrins are freely trafficked within axons of the CST (Andrews et al., 2016). This observation does not seem to be limited to integrins as other transmembrane proteins, such as TrkB and IGF-IR, are also not transported within the entire length of adult axons such as in the CST (Hollis et al., 2009a,b). In the current study however, we show α9-eYFP expression throughout the length of the axonal projections at all time points. As these are early stage developing neurons, we would expect trafficking of growth-promoting integrins to be fully functioning in an anterograde direction encouraging neurite outgrowth and axonal elongation. Indeed, our results demonstrate expression of α9-eYFP from the cell body through to the tip of extending projections.

## Conclusion

In conclusion, although these iPSC-derived hNPCs were not grafted into a SCI model, there is substantial evidence here and from the literature to suggest these cells could be beneficial following SCI. Crucially however, iPSC-derived hNPCs not only present a potential possibility for targeted cell replacement, but they also provide a tool to study integrin adaptation responses in human neurons following exposure to components of the CNS injury milieu. Our data suggests iPSC-derived hNPCs respond to the presence of environmental TN-C by modifying intrinsic integrin activity, a trait previously demonstrated in embryonic DRGs (Condic et al., 1999). Equally, at this developmental state of early postnatal transplantation, our results suggest that overexpression of growth-promoting integrin receptors are not required to induce axonal outgrowth and elongation and that the endogenous levels of integrin may be sufficient for this purpose.

## Author Contributions

MA designed the project. LF and MA carried out the experiments, performed data analysis and wrote the manuscript.

## Conflict of Interest Statement

The authors declare that the research was conducted in the absence of any commercial or financial relationships that could be construed as a potential conflict of interest.

## Supplementary Material

FIGURE S1 Overexpression of α9 integrin-eYFP localizes to vinculin- and actin-rich focal adhesions in hNPCs. The overexpression of α9-eYFP was detected using ICC with an anti-GFP antibody **(A)** within the hNPC projections and co-labeled with the focal adhesion marker, vinculin **(B)** and actin **(C)**; merged in image **(D)**. Integrin expression was observed in the cell body and throughout the length of the projections. At high magnification, α9-eYFP was observed within extended projections in vinculin- and actin-rich areas (inserts of **D**; images **i,ii**). Monochrome images of i and ii are shown in the lower panels. Arrows in **i,ii** indicate localization of α9 integrin to vinculin- and actin-rich focal adhesions; arrowheads in **ii** indicate localization of α9 integrin to an actin-rich focal adhesion site. Scale bar in **(D)** = 25 μm; **(i,ii)** = 5 μm.

FIGURE S2 Changes in endogenous expression of β1 integrin in hNPCs overexpressing α9-eYFP by lentivirus. Endogenous β1 integrin subunit expression was observed in hNPCs using WB, with bands observed at approximately 120 kDa (β1 integrin), analyzed alongside retinal pigmented epithelial 1 (RPE1) cell lysates. Overexpression was confirmed using WB (G) with an anti-GFP antibody, resulting in a band of approximately 140 kDa in lane 2 (LV-α9-eYFP hNPC lysates). Despite unequal protein loading (shown with β-actin at approximately 42 kDa), there is a subtle enhancement of endogenous β1 integrin expression following overexpression of α9 integrin as calculated by the normalized integrated density levels (“Relative β1 integrin expression;” β1 integrin vs. β actin).

FIGURE S3 hNPC grafts were transplanted into the deep layers of the SMC. On-target hNCAM-positive grafts were detected within the deep layers of the SMC **(A)**. This region (cortical layers 5 and 6) can be identified by the presence of TBR1-expressing cells indicated by the white arrows in **(B)**. The cell bolus was detected using anti-HuNu antibody **(C)**; cc, corpus callosum; SMC, sensorimotor cortex; LV, lateral ventricle. Scale bar in **(A–C)** = 500 μm.

FIGURE S4 Transplanted hNPCs show minimal to no overlap with GFAP-positive cells *in vivo*. HuNu-positive hNPCs did not show overlap with GFAP-positive cells following transplantation **(A–D)**. Scale bar **(A,B)** = 250 μm; **(C,D)** = 150 μm.

## Abbreviations

α9: alpha 9 integrin
A-P: anterior-posterior
β1: beta 1 integrin
BCA: bicinchoninic acid
BDNF: brain-derived neurotrophic factor
CNS: central nervous system
CSPGs: chondoitin sulfate proteoglycans
CST: corticospinal tract
Ctip2: chicken ovalbumin upstream promoter transcription factor (COUP-TF) interacting protein 2
D-V: dorsal-ventral
DCX: doublecortin
DRG: dorsal root ganglia
ECM: extracellular matrix
ESCs: embyronic stem cells
eYFP: enhanced yellow fluorescent protein
fGFP: farnesylated green fluorescent protein
GFAP: glial fibrillary acid protein
HEK293T: human embryonic kidney 293 cells expressing SV40 T antigen
hNCAM: human neural cell adhesion molecule
hNPCs: human neural progenitor cells
HuNu: human nuclear antigen
ICC: immunocytochemistry
IHC: immunohistochemistry
iPSCs: induced pluripotent stem cells
M-L: medial-lateral
MBP: myelin basic protein
NSCs: neural stem cells
PLO: poly-L-ornithine
PFA: paraformaldehyde
PKCγ: protein kinase C gamma
RST: rubrospinal tract
SCI: spinal cord injury
Tbr1: T-box brain 1
TN-C: Tenascin-C
Trk/TrkB: tropomyosin receptor kinase/B
VGlut1: vesicular glutamate transporter 1
WB: western blotting
WT: wild type.
