# Supplementary figures and images for: Grafted Human iPSC-Derived Neural Progenitor Cells Express Integrins and Extend Long-Distance Axons Within the Developing Corticospinal Tract
Source: Front Cell Neurosci. 2019 Feb 12;13:26. doi: 10.3389/fncel.2019.00026 (PMC6380224; doi:10.3389/fncel.2019.00026)

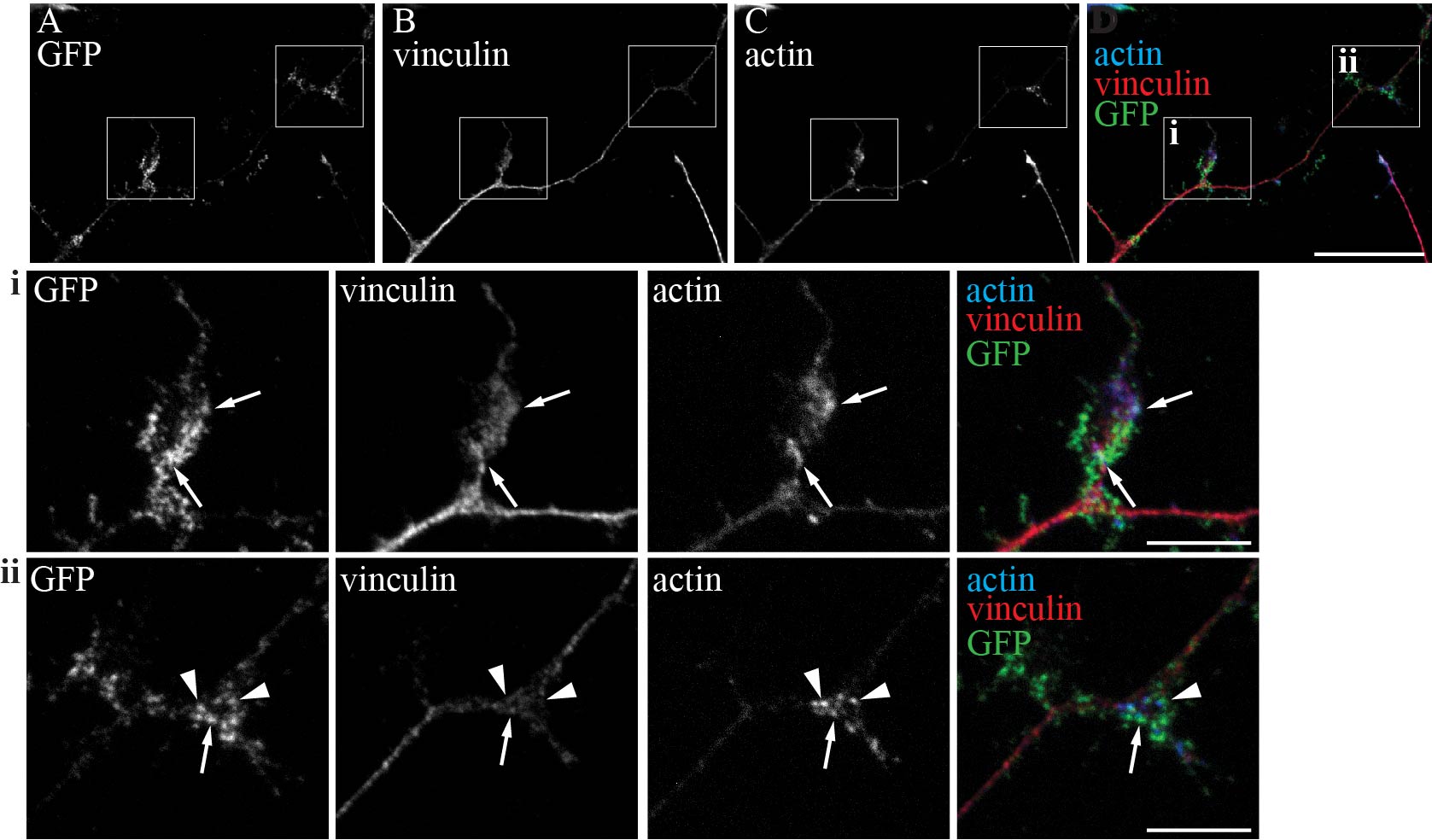

Supplement: FIGURE S1 — Overexpression of α9 integrin-eYFP localizes to vinculin- and actin-rich focal adhesions in hNPCs. The overexpression of α9-eYFP was detected using ICC with an anti-GFP antibody (A) within the hNPC projections and co-labeled with the focal adhesion marker, vinculin (B) and actin (C); merged in image (D). Integrin expression was observed in the cell body and throughout the length of the projections. At high magnification, α9-eYFP was observed within extended projections in vinculin- and actin-rich areas (inserts of D; images i,ii). Monochrome images of i and ii are shown in the lower panels. Arrows in i,ii indicate localization of α9 integrin to vinculin- and actin-rich focal adhesions; arrowheads in ii indicate localization of α9 integrin to an actin-rich focal adhesion site. Scale bar in (D) = 25 μm; (i,ii) = 5 μm. [file Image_1.jpg]

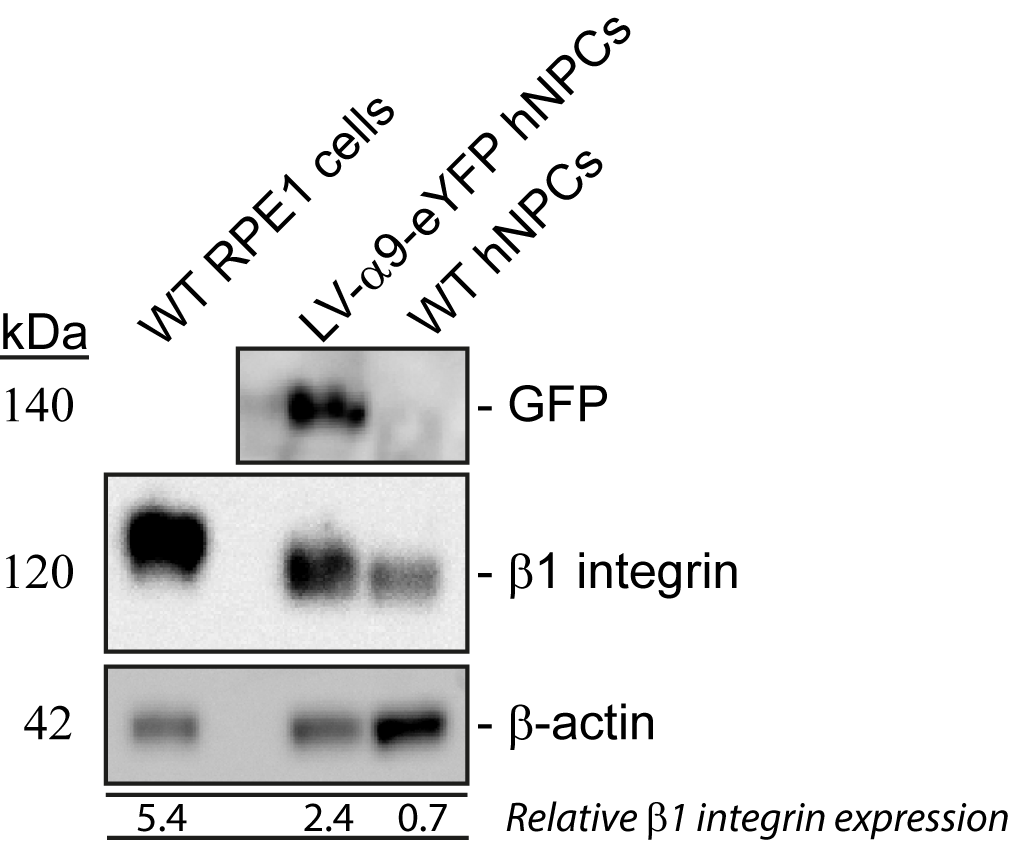

Supplement: FIGURE S2 — Changes in endogenous expression of β1 integrin in hNPCs overexpressing α9-eYFP by lentivirus. Endogenous β1 integrin subunit expression was observed in hNPCs using WB, with bands observed at approximately 120 kDa (β1 integrin), analyzed alongside retinal pigmented epithelial 1 (RPE1) cell lysates. Overexpression was confirmed using WB (G) with an anti-GFP antibody, resulting in a band of approximately 140 kDa in lane 2 (LV-α9-eYFP hNPC lysates). Despite unequal protein loading (shown with β-actin at approximately 42 kDa), there is a subtle enhancement of endogenous β1 integrin expression following overexpression of α9 integrin as calculated by the normalized integrated density levels (“Relative β1 integrin expression;” β1 integrin vs. β actin). [file Image_2.TIF]

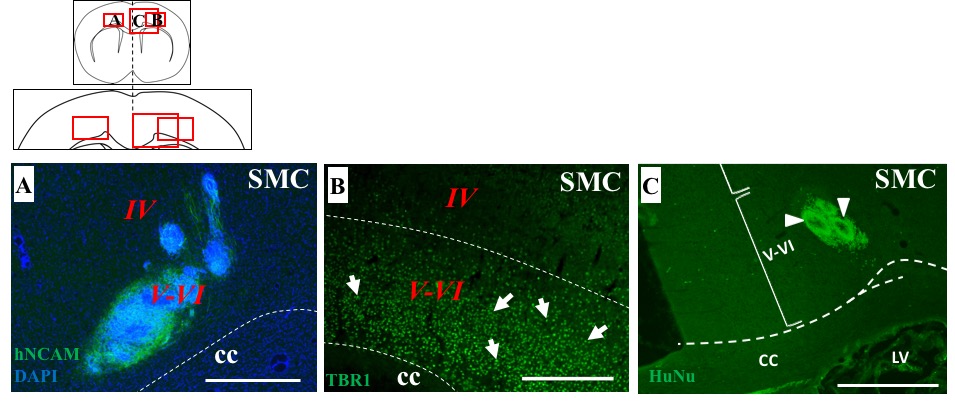

Supplement: FIGURE S3 — hNPC grafts were transplanted into the deep layers of the SMC. On-target hNCAM-positive grafts were detected within the deep layers of the SMC (A). This region (cortical layers 5 and 6) can be identified by the presence of TBR1-expressing cells indicated by the white arrows in (B). The cell bolus was detected using anti-HuNu antibody (C); cc, corpus callosum; SMC, sensorimotor cortex; LV, lateral ventricle. Scale bar in (A–C) = 500 μm. [file Image_3.JPEG]

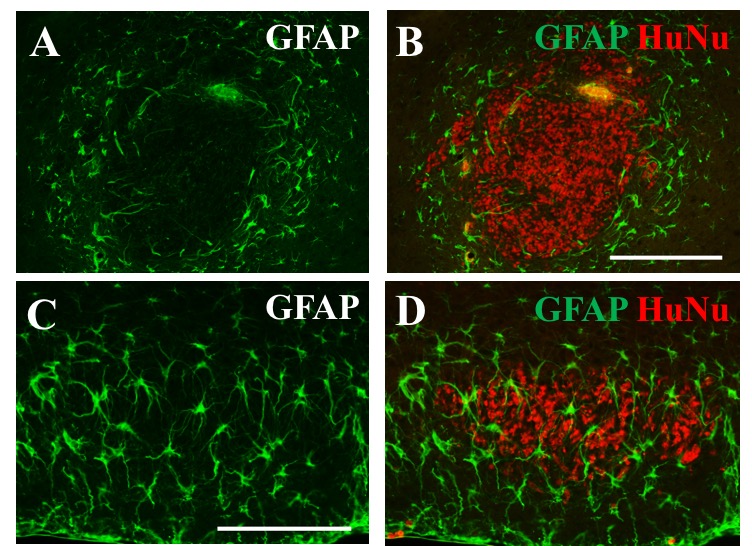

Supplement: FIGURE S4 — Transplanted hNPCs show minimal to no overlap with GFAP-positive cells in vivo. HuNu-positive hNPCs did not show overlap with GFAP-positive cells following transplantation (A–D). Scale bar (A,B) = 250 μm; (C,D) = 150 μm. [file Image_4.JPEG]
